# Identifying Chronotype for the Preservation of Muscle Mass, Quality and Strength

**DOI:** 10.3390/nu18020221

**Published:** 2026-01-10

**Authors:** Roberto Barrientos-Salinas, Norma Dahdah, Jorge Alvarez-Luis, Nuria Vilarrasa, Pablo M. Garcia-Roves

**Affiliations:** 1 Department of Physiological Sciences, Universitat de Barcelona, 08907 Barcelona, Spain; rbarrisa57@alumnes.ub.edu (R.B.-S.); jalvarlu7@alumnes.ub.edu (J.A.-L.); 2 Bellvitge Biomedical Research Institute-IDIBELL, Hospitalet de Llobregat, 08907 Barcelona, Spain; nuria.vilarrasa@ub.edu; 3 Faculty of Sport Sciences, Antonine University, Hadat-Baabda, Lebanon; dahdahnorma@gmail.com; 4 CIBER de Diabetes y Enfermedades Metabólicas Asociadas (CIBERDEM), Instituto de Salud Carlos III, 28029 Madrid, Spain; 5 Department of Endocrinology and Nutrition, Hospital Universitari Bellvitge Hospitalet de Llobregat, 08907 Barcelona, Spain; 6 CIBER de Obesidad y Nutrición (CIBEROBN), Instituto de Salud Carlos III, 28029 Madrid, Spain

**Keywords:** chronotype, circadian rhythm, clock genes, muscle mass, muscle strength, muscle health, obesity, sarcopenia, sleep, hormonal fluctuations

## Abstract

Chronotype, an individual’s preferred timing of sleep and activity within a 24 h cycle, significantly influences metabolic health, muscle function, and body composition. This review explores the interplay between circadian rhythms, hormonal fluctuations, and behavioral patterns—such as nutrition timing, physical activity and sleep quality—and their impact on muscle mass, strength, and quality. Evening chronotypes (ETs) are consistently associated with poorer sleep, irregular eating habits, reduced physical activity, and increased risk of obesity, sarcopenia and metabolic disorders compared to morning types (MTs). At the molecular level, disruptions in circadian clock gene expression (e.g., BMAL1, PER2, CRY1) affect protein synthesis, insulin sensitivity, and energy metabolism, contributing to muscle degradation and impaired recovery. The review highlights critical components—targeting chrono-nutrition, sleep quality, and exercise timing—to align lifestyle behaviors with circadian biology, thereby preserving muscle health and improving overall metabolic outcomes.

## 1. Introduction

Human metabolism is complex; even though it is at the center of all processes delineated by an individual’s genome, it is strongly affected by external factors. Metabolic regulations dictate the physiological responses of an organism with the purpose of reaching optimal responses to internal and external stimuli. Those regulatory pathways are stimulated and defined by the interaction of the organism with its surroundings, including environmental risks and the habits of an individual, such as sleeping hygiene, nutrition, level of activity and much more [1]. In this review, the discussion will revolve around specific metabolic regulations effected by nutrition and training related to chronotype that lead to alterations in metabolic processes. One of the most crucial roles of human metabolism is to transform the chemical energy obtained through the digestion of food into cellular energy, allowing system function, involving processes relevant to this review article such as protein synthesis, cellular proliferation, bodily movement and so on [2].

Excessive energy intake through a permissive food environment marked by the type, quantity, quality and timing of food, coupled with low energy expenditure—a low level of physical activity or even sedentarism—marks an energy imbalance, meaning excess energy has to be stored. When this occurs constantly for a prolonged period, excess energy turns into excess fat accumulation, leading to people being overweight or living with obesity [3].

Due to obesity, the increase in muscle mass is mostly neglected, and the focus is shifted towards the increased fat mass and its deleterious effect on human health. However, obese people in comparison to normal-weight people have higher total fat-free and higher muscle mass [4,5], but lower muscle quality (strength per unit of muscle). This is a hidden health condition that is commonly overlooked, undiagnosed, and associated with poor health outcomes [6]. Factors such as fat infiltration (myosteatosis) negatively impact muscle quality and strength. The lack of muscle mass and strength, known as sarcopenia, when coexisting with excess adiposity, results in a modern condition known as sarcopenic obesity, commonly seen in older adults and associated with increased health risks [7]. In addition to the increased risk of sarcopenia due to obesity, the same risk exists when weight loss is substantial in a short period of time. Thus, the need for weight loss strategies that maintain muscle mass along with muscle strength has increased and has been proven challenging [8].

Several approaches have been proposed as potential strategies for weight loss, including nutritional interventions, pharmacological treatments, and bariatric surgeries. However, these protocols may negatively impact the preservation of skeletal muscle, potentially limiting their long-term efficacy [9]. Among the factors that remain insufficiently explored, fluctuations in circadian rhythms and their influence on systemic homeostasis and its impact on metabolism, have emerged as a compelling variable. This represents a window of opportunity for a more integrative management approach—one that considers the specific rhythmic variations in each individual framed within their lifestyle and preferred resting-active timing in accordance with the light/dark cycle in a 24 h day, known as a chronotype [10].

Chronotypes display unique characteristics and behavioral patterns that can influence muscle health—either positively or negatively—and, by extension, overall well-being.

This study aims to deepen our understanding of chronobiology and the specific traits associated with different chronotypes with the goal of providing strategies to support muscle health and adjust behavioral patterns that may negatively impact muscle preservation and body composition. These strategies are crucial for preserving muscle during weight loss.

## 2. Circadian Rhythm

Humans, similarly to most living organisms, have an internal clock that generates circadian rhythms involved in the regulation of gene expression, metabolism and behavior [11,12,13]. A key structure to circadian rhythms, its central clock located in the hypothalamus, is known as the suprachiasmatic nucleus (SCN) and it communicates via several pathways with multiple structures in the brain and peripheral organs [14]. The SCN gathers information from sensory inputs to synchronize the internal and external cycles. As a result, the SCN projects the timing to the other structures, creating a “temporal framework” of physiological and behavioral processes [15]. The SCN regulates this process through dual output mechanisms: direct neural pathways projecting to adjacent hypothalamic and thalamic nuclei, which act as relays to control various physiological functions and behaviors; and endocrine output signals using non-synaptic communication to synchronize peripheral clocks throughout the body [16,17].

External cues such as light perception, feeding patterns and environmental conditions like temperature fluctuate in a 24 h cycle creating a physiological challenge to which homeostatic controls have to respond efficiently for optimal survival. A critical function of the circadian rhythm is to detect these repeated patterns of external stimuli, adapt to them and finally gain the ability to anticipate them [18].

Daily patterns such as hormonal fluctuation have implications for many physiological functions throughout the day, which are directly involved with metabolic health. Alterations in the synchronicity of the circadian system have been correlated with glucose tolerance and diminished insulin sensitivity, heightened levels of proinflammatory cytokines, elevated arterial blood pressure, and decreased energy expenditure that may affect the cardiovascular system and are connected to an elevated susceptibility to metabolic disorders [19,20].

Disruptions to the body’s internal clock can lead to disturbances in the sleep–wake cycle and abnormalities in hormone regulation, blood pressure, heart rate, and other vital processes. Long-term disturbances have been linked to the development of chronic degenerative diseases, including cardiovascular diseases, metabolic disorders, tumors, neuropsychiatric conditions, and so on [21].

At the molecular level, clock genes serve as the basis of an intracellular timekeeping system, comprising interlocking feedback loops composed of cycling gene products that control transcription by means of negative and positive regulation of clock genes and proteins [22]. Post-transcriptional regulation of clock proteins plays an important role in rhythm generation and the synchronization of the biological clock to daily environmental fluctuations [23].

### Clock Gene Expression

Environmental factors affect the central circadian clock, which transmits the rhythms to the peripheral clocks and the clock-controlled genes. Light, or the daily light–dark cycle, is the primary environmental cue that sets the 24 h oscillations of circadian rhythms in the organism. Another major influencer on these 24 h oscillations is food intake, more specifically, the feeding-fasting cycles in addition to the composition of food intake (the macronutrients) [11,24]. Circadian oscillations are generated at a cellular level, a core mechanism found in most cells and known as the cell-autonomous transcriptional-translational feedback loop (TTFL) [25,26].

Circadian locomotor output cycles kaput (CLOCK) and brain and muscle ARNT-like protein-1 (BMAL1) are considered core components of the mammalian circadian clock. BMAL1 heterodimerizes with CLOCK to form the BMAL1:CLOCK complex, which can then act as a transcriptional factor regulating genes involved in circadian rhythms by binding to specific cis-acting DNA sequences or E boxes. The BMAL1:CLOCK complex regulates a wide range of circadian outputs via the activation or repression of the transcription of genes encoding protein synthesis participating in circadian rhythms [27,28].

**Figure 1:**
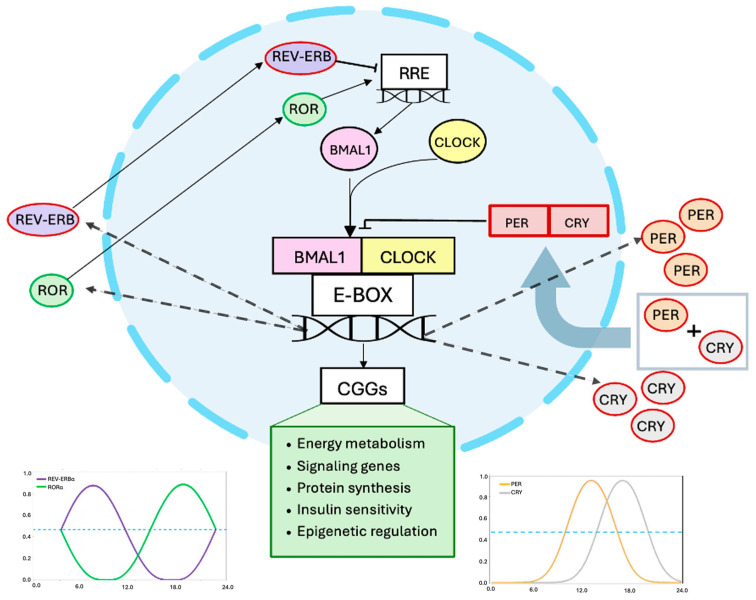
Clock genes regulate the function of the mammalian circadian rhythm. BMAL1:CLOCK activates PER and CRY transcription via E-box elements; PER/CRY complexes inhibit BMAL1:CLOCK, forming a negative feedback loop. BMAL1 expression is further regulated by REV-ERB and ROR through RREs. These interlocked loops drive rhythmic gene expression. Chronotype-specific alterations in the amplitude or phase of these components—e.g., delayed PER/CRY accumulation in evening types—can disrupt clock-controlled outputs and metabolic homeostasis.

The transcription factors BMAL1 and CLOCK form heterodimers, which bind to the E-box located in the promoter regions of the period (per) and Cryptochrome (cry) genes to promote the production of PER and CRY proteins. When they accumulate to a certain level in the cytosol, PER and CRY translocate to the nucleus to inhibit the activity of the BMAL1:CLOCK complex, thus repressing their own expressions [29]. In addition, a secondary feedback loop involving the nuclear receptors ROR and REV-ERB modulates BMAL1 transcription by activating or repressing its expression through direct binding to ROR/REV-ERB response elements (RREs) within the BMAL1 promoter [30]. These core clock components regulate hundreds of other genes, called clock-controlled genes (CCGs) in a circadian manner (Figure 1). Generally, core clockwork mechanisms exist in most tissues and cells of the body, but the expression of CCGs varies by cell type [21].

## 3. Chronotype

“Chronotype” is a term used to describe an individual’s preferred sleep–wake (resting-active) timing in accordance with the light/dark cycle in a 24 h day. A chronotype is influenced by external factors such as light exposure and food intake and also by internal factors such as genetics. Furthermore, an individual’s chronotype reflects their diurnal preferences and controls physiological processes and behavior [31,32].

In a 24 h day, humans are physiologically programmed to be alert, active and in a fed state around two-thirds of the day, and the remaining one-third, they sleep and are in a fasting state. Biologically, humans are diurnal, which means they are active during the day and resting, more specifically asleep, at night. However, due to personal preferences and social lives, environment, work schedules and shifts, new dietary habits, increased caffeine consumption, and the exposure to artificial light from computer screens and smartphones, a shift in the “normal diurnal behavior” gradually occurred, leading to the familiarization with the term “chronotype” [32,33,34,35,36].

Three main chronotypes are distinguished: morning-type (MT), evening-type (ET) and neither-type (NT) [10,11,32,37]. MTs are individuals that follow the diurnal timing; they wake up early and achieve peak mental and physical performance early in the day, and they sleep early. On the other hand, for ET individuals, their peak mental and physical performance takes place in the evening and thus their sleeping time is delayed. About 40% of the adult population is classified in one of the two extreme groups, while 60% are NT [31,32]. Time of food intake and the “highest caloric intake” have also been used to determine a person’s chronotype. MTs have their highest caloric intake—represented by 20% of total daily intake—early in the day, while ETs have it later in the day, closer to nighttime [38]. Finally, the most common chronotype NT, also known as intermediate-types, are individuals that have no strong circadian preference; they show intermediate characteristics [39].

Daytime performance is also influenced by chronotype, with morning types (MTs) exhibiting superior outcomes in the early afternoon, whereas evening types (ETs) demonstrate enhanced performance in the late evening [40]. MTs tend to exhibit earlier peaks in BMAL1 and PER2 expression, which may align with better neuromuscular readiness and metabolic efficiency earlier in the day. On the other hand, ETs show delayed expression peaks, potentially explaining their superior performance in the late afternoon or evening hours [41]. Evidence suggests that depending on the phase of expression of some clock genes, such as BMAL1, PER2, and NR1D1, the training response could be affected, explaining the peak levels of performance occurring in the late afternoon/evening compared with the morning hours, as reflected by better results in different parameters, such as power output, force production, and maximal oxygen uptake (VO_2_ max). However, morning exercise might offer better metabolic benefits for disorders like diabetes, suggesting that exercise timing matters for health [42].

### 3.1. Chronotype and Body Composition

The circadian clock significantly influences body composition by regulating metabolism, eating patterns, and energy storage and expenditure. Disruption of circadian rhythms, such as erratic eating patterns or shift work, can lead to metabolic disorders, obesity, and changes in body composition. Conversely, optimizing eating patterns aligned with circadian rhythms while maintaining regular eating cycles can have a positive impact on body composition [43].

It has been established that the quantity and quality of food in meals are not enough as determinants of metabolic health. A study performed in 2014 [44] on adolescents has revealed that ETs had a small but significantly increased body mass index (BMI) in comparison to MTs. They also documented different dietary behaviors for ETs, highlighted by inadequate daily intake of fruits and vegetables, increased consumption of unhealthy snacks and increased consumption frequency of nighttime caffeine [44]. It has been shown that people who have a higher caloric intake during the final hours of the day, when the increase in melatonin secretion begins (which usually occurs 2–4 h before falling asleep), show an increase in fat storage, which is associated with a higher BMI [45]; thus, increasing the risks of obesity and cardiovascular diseases [39,46]. Another study performed in Japan [47] with female students aged 18–22 years as participants has observed a significantly lower skeletal muscle index (SMI) and significantly higher body fat percentage in ETs compared to non-ETs. No significant differences in total energy intake between MTs and ETs were recorded; however, the distribution of nutrients differed greatly, with ETs tending to have irregular eating patterns along with skipping meals and consuming most of their calories in the evening and/or at nighttime. A connected study has also reported different patterns in behavior related to physical activity; ETs have the tendency to be significantly less active than MTs when excluding academic and social obligations [48]. When chronotype and metabolic health were tested in Korean adults aged between 40 and 69 years, out of 1620 individuals enrolled in the study, 5.8% were considered as ETs and they showed increased prevalence of diabetes, metabolic syndrome and sarcopenia along with an increased fat mass in women and decreased lean mass in men [31]. The misalignment between internal and external rhythms is greatest in ETs [31]. On weekdays, ETs tend to have sleep debts due to insomnia and other sleeping disorders, which leads to extended sleep on weekends to compensate [49]. Irregularity in sleeping schedule, independently of sleeping duration, was associated with obesity [50]. In addition, the circadian misalignment was linked to higher postprandial glucose and insulin levels, lower leptin levels [51] and a more pronounced insulin resistance and inflammatory profile [52]. By contrast, peripheral insulin sensitivity was found to be greater for MTs who also revealed decreased homeostatic model assessment of insulin resistance (HOMA-IR), adipose insulin resistance (Adipose-IR) and fasting insulin levels [53]. This finding is in correlation with other studies that revealed increased visceral fat and abdominal obesity in ETs compared to MTs [54,55].

The increased risk for developing metabolic disorders (metabolic syndrome, obesity, diabetes, etc.) in case of misalignment between internal and external chronotype has been well documented through the observation of parameters such as BMI and fat mass percentage. However, the measurement of markers related to skeletal muscle mass in association with chronotype alignment was mostly tackled by scientists interested in physical activity and performance, which will be discussed in a later section [56].

### 3.2. Chronotype—Muscle Health and Protein Synthesis

Skeletal muscle is a tissue that plays a central role in metabolic function, daily activity performance, and the prevention of many chronic diseases [57]. The importance of preserving muscle health is growing in the clinical field and has become a current challenge in combating various weight loss methods, which often involve a significant loss of muscle mass (25% to 29%) and strength [58], increasing the risk factors for morbidity and mortality in the older adult population.

The preservation of muscle health requires the integrated assessment of three fundamental domains: functional status, which reflects the normative development of muscle mass and strength; muscle performance, which evaluates the adequacy of muscle capacity to meet the demands of daily activities; and tissue composition, which provides insight into the efficiency of protein synthesis and the maintenance of muscle mass. The optimal development and maintenance of these domains is contingent upon overall muscle health, which is modulated by both external factors—such as nutritional intake, physical activity, and the temporal organization of daily behaviors—and internal factors, including endocrine regulation [59].

Skeletal muscle protein metabolism is regulated by mechanical, nutritional, and hormonal inputs. Testosterone and IGF-1 enhance anabolism through activation of the Akt/mTOR and PI3K/Akt/mTOR pathways, whereas cortisol promotes catabolism via protein degradation mechanisms [60]. Consequently, behavioral and lifestyle factors—including poor sleep quality, inadequate dietary intake, altered meal timing, sedentary behavior, and psychological stress—may compromise muscle health by impairing protein synthesis, ultimately reducing muscle function, composition, and performance [61].

Individual chronotype patterns may significantly influence muscle health and protein synthesis. For instance, the evening chronotype (ET) has been associated with lower levels of physical activity, greater sedentary time, poor sleep quality, insulin resistance, and increased risk of cardiovascular disease, all of which contribute to reduced muscle protein synthesis and loss of muscle mass [62,63,64]. Declines in muscle mass are typically accompanied by reductions in muscle strength, predisposing individuals to sarcopenia and frailty [65,66]. Moreover, muscle mass loss is closely linked to insulin resistance, metabolic syndrome, and age-related sarcopenia [67].

Sarcopenic obesity, defined in Section 1 of the Introduction, is a driver to find new strategies for weight loss, preventing decline in muscle mass and muscle strength [6]. The key to all strategies should be to maintain a healthy skeletal muscle turnover—the balance between muscle protein synthesis and muscle protein breakdown—through nutritional alterations, physical activity stimulation and hormonal regulations that promote protein synthesis [68].

### 3.3. Chronotype and Hormonal Fluctuation

Hormonal fluctuations are directly influenced by the internal body clock. Altered hormonal rhythms imply broad physiological and psychological actions, including effects on metabolism, glucose utilization, muscle mass and strength, adiposity, physical performance and well-being. People with different chronotypes, through their behaviors, could affect hormonal patterns and, hence, can impact health conditions [69,70]. For example, insulin sensitivity to glucose metabolism is lower in the afternoon, and consequently, when the highest caloric intake for ETs is in the afternoon or evening time, it results in a greater increase in plasma glucose and glucose intolerance compared to when the meal is in the morning [19].

Moreover, and besides the metabolic alterations leading to decreased glucose tolerance, eating late leads to a lower resting-energy expenditure and lower thermal effect of food, and finally, the usual cortisol fluctuations during a 24 h cycle are blunted [71].

Melatonin has been found to be at higher levels during the daytime of ETs. Such alterations of the melatonin secretion pattern can directly affect sleep quality, which is closely linked to health and performance. Poor sleep quality has effects on skeletal muscle homeostasis and glucose throughout the body that predispose people to various disease states, such as obesity, insulin resistance, and type 2 diabetes, and increased risk of all-cause mortality [72]. Endocrine function is greatly affected by poor sleep quality, causing alterations in the concentrations of appetite hormones such as leptin and ghrelin, which influence the feeling of hunger and satiety. In addition, the secretion of steroid hormones is affected by sleep, for example, increased plasma cortisol concentrations.

Conventionally, melatonin secretion has nocturnal peaks where it starts rapidly rising 2 to 3 h before sleep onset (evening) and rapidly falling in the morning [73,74]. There have been several studies that aimed at disrupting this pattern or showing differences in melatonin secretion under certain circumstances. Interrupting nighttime through light exposure has frequently been studied for its role in inhibiting melatonin secretion [75,76]. A shift in the sleep/wake cycle induced an increase in melatonin levels in the daytime [75]; in addition, short afternoon naps had the same effect of increased melatonin levels in the daytime [77]. Neuropathological disorders have been found to alter melatonin levels [78,79,80,81].

Cortisol is an important regulator of energy homeostasis. In response to stress in the form of perceived danger or acute inflammation, cortisol is released from the adrenal gland, rapidly mobilizing energy from glycogen, fat and proteins. In the case of inflammation, mobilization of proteins/amino acids is critical for the rapid synthesis of acute phase reactants and an efficient immune response to infection. While adaptive in response to infection, chronic mobilization can lead to a profound depletion of energy stores. Skeletal muscle represents the major body store of protein and can become substantially atrophied under conditions of chronic inflammation [82].

Furthermore, cortisol awakening response has been shown to peak around 30 min after waking up. This response varies between populations, influenced by light exposure, age, sex, health and stress. Evening-types do not demonstrate such a peak in cortisol levels in the first hour of being awake [82]. These alterations in the hormonal environment induced by lack of sleep can be a catalyst that sustains alterations in skeletal muscle metabolism by decreasing the rate of muscle protein synthesis resulting in a loss of muscle mass and muscle strength affecting functional outcomes [13].

Changes in hormonal secretion have been explained through studies that investigate the expression and regulatory roles of circadian genes. For example, in MTs, rising cortisol levels during the first hours of the day enhance the transcriptional activity of genes such as BMAL1 and PER2, aligning metabolic processes with the active phase of the day [20]. Conversely, the timing of food intake for the ETs, particularly when melatonin levels increase, disrupts the rhythmic expression of genes like CRY1 and PER1, impairing glucose tolerance and lipid handling. These feeding transitions actively reprogram gene expression in key metabolic tissues, reinforcing the role of nutrient timing as a modulator of circadian gene dynamics and overall metabolic homeostasis [83]. Similarly, groups exhibiting intermediate chronotype behavior, such as NTs, tend to display patterns more closely aligned with morning types than evening types, both in terms of dietary behavior and metabolic health, suggesting a more stable or less phase-delayed expression of genes such as BMAL1 and PER2 [84].

These differences related to metabolic response could be explained by the role of circadian clock genes in regulating energy homeostasis, given that, as mentioned previously, their expression patterns vary depending on the time of day. For instance, in white adipose tissue (WAT), transcriptional activity of the BMAL1/CLOCK complex, in a peripheral expression, governs key metabolic processes including lipogenesis, lipolysis, and β-oxidation, thereby influencing adipocyte proliferation and differentiation [85]. Consistent with this, studies in murine models have shown that deletion of Bmal1 disrupts the expression of central adipogenic regulators such as peroxisome proliferator-activated receptor gamma (PPARγ) and CCAAT/enhancer-binding protein alpha (Cebpα), leading to impaired adipose tissue functionality [86]. Along the same lines, a dysregulation at crosstalk levels has been described in mice, where an early nocturnal meal skipping—analogous to breakfast omission in humans—induces a misalignment of peripheral circadian clocks, promoting fat storage by the increase in lipogenic activity [87]. This study, by Yoshida et al. (2012), showed that mice subjected to early nocturnal fasting (ZT12–18) exhibited altered acrophases of core clock genes such as Bmal1, Per1, and Cry1 in liver and adipose tissue accompanied by upregulation of lipogenesis-related genes including Sterol Regulatory Element-Binding Protein 1c (Srebp-1c) and Fatty Acid Synthase (Fasn), despite an isocaloric intake [87].

## 4. Chrono-Strategy to Preserve Muscle Health

Emphasizing the role of skeletal muscle tissue in maintaining overall health, it is important to highlight certain behavioral patterns influenced by factors regulating muscle metabolism throughout the day, such as chronotype. These personal daily patterns can alter muscle function and health, potentially leading to the loss of strength and muscle mass. The regulation of skeletal muscle mass can be disrupted by alterations in the factors that govern protein metabolism within skeletal muscle [61]; this could be due to the following: 1—lack of physical activity, eliminating the mechanical factor of contraction, which favors muscle synthesis; 2—a poor diet, favoring foods rich in fats and sugars and low in proteins; and/or 3—poor sleep quality, favoring the degradation of muscle proteins and increasing cortisol [60].

Moreover, it has been shown that ETs have a poor sleep quality and are prone to sleep deprivation [88,89], which is associated with induced anabolic resistance and a procatabolic environment. The rise in cortisol levels promotes catabolism by activating key muscle protein degradation pathways [19]. This condition may exacerbate the anabolic resistance, especially in people with obesity, which may affect body composition (e.g., high fat mass and low lean mass), muscle weakness (associated with lower lean mass), physical disability and poor quality of life, especially in older individuals with obesity [90].

**Figure 2:**
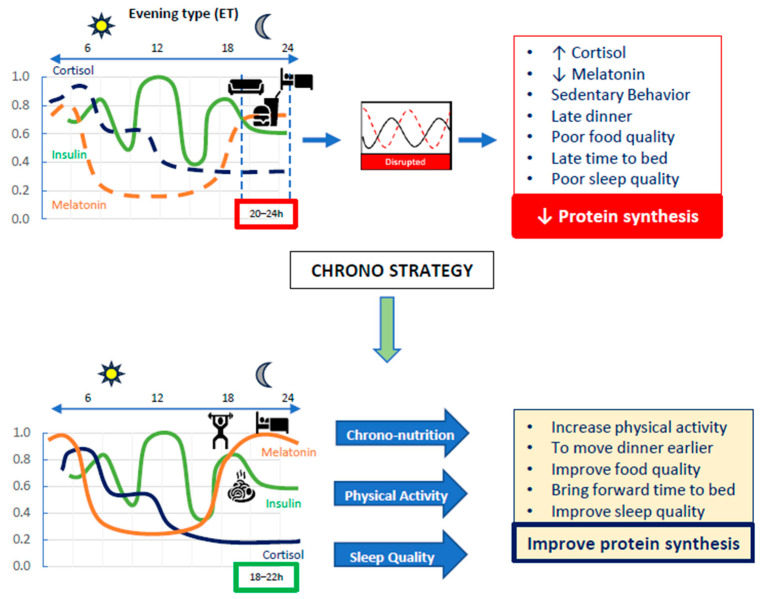
Representative image—Chrono strategies to favor protein synthesis. Evening chronotypes (ETs) are commonly associated with low levels of physical activity, sedentary behavior, poor sleep quality, and unhealthy dietary patterns. These factors contribute to hormonal imbalances—particularly in insulin, cortisol, and melatonin—which impair protein synthesis and accelerate muscle mass loss. Therefore, alignment of these patterns to the circadian clock could help to preserve muscle mass through an increase in protein synthesis. Dotted lines indicate disruption.

If ET is considered the chronotype with the most adverse outcomes, what would happen if we could modify some of these behaviors to positively influence muscle health and, consequently, overall health? (Figure 2).

### 4.1. Targeting Chrono-Nutrition

The total energy balance and the associated alterations in body weight are not components of a straightforward simple equation. For example, several studies have demonstrated that high-fat diet protocols induce a weakened diurnal rhythm and an extended circadian cycle, mechanisms that ultimately contribute to insulin resistance, local inflammation, and a disruption in the secretion balance of adipokines, such as leptin and adiponectin [91,92]. Nutritional and health status is complex; many factors interact, such as hunger, satiety, food availability and consumption, the environment and social factors. The energy consumed is not solely defined by the quality and quantity of the meal. There is a new focus on nutritional chronotype: the time at which the food is consumed and its nutritional value in accordance with other physiological indicators such as sleep and physical activity. For further clarification, nutritional chronotype is marked by the frequency, time of the meal (beginning and end), its nutritional value, the regularity and the scheduling of the daily intake in relation to the events of daily life such as work, exercise, leisure, sleep and social relationships, among others [93]. Several studies have mentioned metabolic health benefits—primarily determined by body weight, BMI, and body composition—in case of alignment between the external clock and the internal clock [38]. Hence, it has been shown that alignment between the external clock—translated as the timing of the highest caloric meal consumed—and the internal clock—translated as the individual’s chronotype—is associated with decreased BMI and fat mass index (FMI) along with increased fat-free mass index (FFMI) [38].

So, switching the time of meal consumption, most importantly the highest caloric meal consumption, to earlier in the day could positively affect metabolic health. These actions should respect the typical insulin fluctuation in a 24 h cycle in order to enhance insulin sensitivity, leading to a better tolerance to glucose.

### 4.2. Targeting Sleep Quality

It has been established that certain hormones enhance protein synthesis by activating anabolic signaling pathways or by suppressing the expression of genes associated with protein degradation [60]. Experimental evidence further suggests that behavioral patterns such as changes in sleep duration can significantly influence muscle metabolism by directly interacting with the expression and function of the circadian clock [8].

Sleep is essential for muscle health, yet it is increasingly disrupted in modern society due to 24 h exposure to artificial light, unrestricted access to energy-dense snacks, social engagements, and low levels of physical activity. These factors interfere with normal circadian rhythms, carrying profound implications for various physiological and metabolic processes.

Sleep restriction impairs skeletal muscle glucose homeostasis and alters the regulation of appetite-related hormones such as ghrelin and leptin. Additionally, sleep deprivation leads to elevated secretion of steroid hormones, notably increasing plasma cortisol concentrations. These hormonal disturbances may act as catalysts for impaired skeletal muscle metabolism, resulting in decreased rates of muscle protein synthesis, loss of lean mass, and concomitant reductions in muscle strength and functional capacity [85].

Consistent, high-quality sleep is essential for optimal muscle synthesis and recovery, as deep sleep stages—particularly slow-wave sleep—trigger the release of growth hormone, which stimulates muscle protein synthesis and tissue repair. Several studies show that sleep deprivation impairs anabolic signaling (e.g., mTOR), reduces testosterone, elevates cortisol, and alters muscle gene expression, all of which hinder muscle growth and increase protein breakdown. To support muscle health, adults should maintain a regular sleep–wake schedule, avoid late-night training and screen exposure, and consume protein-rich meals earlier in the evening to enhance overnight recovery [13,90].

### 4.3. Targeting Physical Activity

Exercise plays a pivotal role in modulating circadian rhythms and chronotype-related health outcomes and also plays a key role in stimulating muscle protein synthesis, promoting hypertrophy, and enhancing adaptation to physical stress. Resistance training, in particular, contributes significantly to increases in both muscle mass and strength, thereby supporting overall muscle health, while aerobic and high-intensity interval training improve sleep quality and facilitate circadian adjustment under disrupted light/dark schedules. Notably, engagement in regular physical activity during midlife has been associated with a reduced risk of sarcopenia and serves as a positive predictor of muscular strength and physical performance in older age [94]. Time of exercise, particularly in the midday–afternoon or evening, is consistently associated with cardiovascular benefits, including reductions in blood pressure, heart rate, and cardiometabolic risk. Moreover, exercise enhances glucose and lipid metabolism, increases insulin sensitivity, and mitigates metabolic disease risk, partly through its regulatory effects on circadian rhythms [21].

Resistance training enhances muscle size, strength, and metabolic capacity through mechanical loading and intracellular signaling pathways. Skeletal muscle functions as a circadian tissue, with clock components and systemic hormonal rhythms modulating metabolism, protein synthesis, and exercise responsiveness. Training outcomes vary by time of day: evening sessions favor hypertrophy and anabolic signaling via pathways like ribosomal biogenesis and mTOR, while morning sessions enhance mitochondrial and autophagy-related pathways. These findings highlight the importance of exercise timing and support incorporating chronobiological principles into training prescriptions to optimize muscle adaptation and individualized strategies [95,96].

At the molecular level during exercise, some modulators of energy homeostasis, such as AMPK, are overexpressed and regulate the expression of PER and CRY, thereby directly influencing metabolic processes and hormonal responses that impact exercise performance [97]. Furthermore, the role of PER2 extends beyond circadian regulation and has been implicated in the modulation (inhibition) of the mammalian target of rapamycin complex 1 (mTORC1), a central regulator of protein synthesis and glucose and lipid metabolism [98]. PER2 directly promotes the recruitment of the protein hamartin (TSC1) to the mTORC1 complex, thereby inhibiting its activity. Consequently, beyond its canonical role in the circadian cycle, PER2 has emerged as a key determinant of exercise physiology and performance [99]. Regarding the interaction between other circadian clock genes, it has been described how BMAL1 modulates mTORC1 activity too. BMAL1 knockout in mouse models reveals mTORC1 hyperactivation through enhanced phosphorylation of downstream targets such as Ribosomal Protein S6 Kinase Beta-1 (S6K1) and Eukaryotic Translation Initiation Factor 4E-Binding Protein 1 (4EBP1), highlighting the negative regulatory role exerted by BMAL1 on this signaling pathway [100].

## 5. Conclusions

The evidence reviewed demonstrates that chronotype is a key determinant of muscle and metabolic health. Alterations in circadian rhythms and clock gene expression directly affect protein synthesis, insulin sensitivity, and body composition, favoring the development of obesity, sarcopenia, and sarcopenic obesity—particularly in individuals with an evening chronotype. These individuals often present poor sleep quality, irregular eating habits, and lower physical activity, which increase the risk of muscle mass and strength loss.

Therefore, it is essential to implement integrated chronobiological strategies—such as optimizing meal timing (chrono-nutrition), improving sleep quality, and synchronizing exercise with circadian rhythms—to preserve muscle mass, quality, and strength. Aligning daily behaviors with circadian biology not only helps maintain muscle health but also enhances metabolic outcomes and reduces the risk of chronic diseases.

## Data Availability

No new data were created or analyzed in this study. Data sharing is not applicable to this article.
